# Annals of Quackery—IV

**Published:** 1910-07-09

**Authors:** 


### SPECIAL ARTICLE.

### ANNALS OF QUACKERY—IV.

Two notorious quacks who flourished a hundred years ago were known as the "Doctors" Jordan. In Cannon Street Road, within a few doors of the turnpike, stood a shop parlour then but recently vacated by a mangle and its industrious proprietor. This shop appeared to the Jordans, who at that time were hawkers of lead pencils, in every particular calculated for study and to improve them in their about-to-be-adopted profession, and consequently having struck a bargain they took possession of the mangling shop. Blue bottles were put in the windows, gallipots on the counter, and they published in large placards and handbills that they had become suddenly inspired by a "golden dream." Strange as it may appear, many people believed in the imposition, and before long the "doctors" became possessed of a gig, in which, with a livery servant, they used to tour the provinces. They professed to cure all diseases, but more especially consumption, with their "Balm of Rakasiri," five "family" bottles of which—presumably the quantity required to effect a "cure"—cost £8 5s. They were particularly heartless swindlers, as one may well imagine, while some idea of the kind of education they had received may be obtained from the letter which one of them wrote to the editor after the attack appeared in the *Medical Adviser*. "I never hokd pecels," says the letter, "I only tuk orders for 'em, and even if I did it is no affere of yours. I got my bred hunestly. . . . You wood do better to tack the regular doctors than me for they har proper rogues every won on 'em." The attacks evidently did these "doctors" some damage, for they were hooted in many of the places they subsequently visited.

Another famous quack was Mrs. Johnston, who pushed herself from poverty and obscurity into an elegant house in the City Road. She is dubbed the "child-killer," as her chief medicine was a soothing syrup containing opium. Apparently she was patronised by the rich as well as the poor, for speaking of her elegant patrons the *Medical Adviser* says, "These women are fond of quiet nights while in bed and idle gadding in the daytime." Among the most interesting of the quacks of a century ago was "Doctor" Macdonald. He was originally a cowherd, and although his life was unenlightened by learning and unbiassed by virtue he managed to get himself into a fine house in the Kent Road, and according to account "to live like an alderman." Like other quacks he began by privately administering his nostrums for various disorders. He resorted to alehouses, and by getting into conversation with his pot companions persuaded one or two occasionally to take his medicine. By economy, industry, and perseverance he crept on from penny pills until he became "Doctor Macdonald of the Kent Road." He established a kind of waiting-room in which the poor people assembled, and from each person who came his man exacted one penny for a ticket of admission. He employed four or five people to place themselves at different parts of the room, and there for the space of three hours speak of the marvels the "doctor" had wrought. He soon saw that a little advance upon his admission tickets would make no difference to his customers, and he therefore increased the sum to threepence. The system of his consultation was somewhat out of the ordinary. At eight o'clock he admitted the threepenny patients until ten o'clock, when his wife would call out at the foot of the stairs, "Doctor, it is ten o'clock, and the half-crown patients are all waiting." The half-crown patients were then admitted until twelve o'clock, when his wife would call out, "Doctor Macdonald, the half-guinea patients are all ready." Apparently he continued to prosper, for when his turn was reached in the *Medical Adviser* he had a house fitted up as an infirmary, "where he kills by wholesale. He charges the inmates so much for curing and for board and lodging, doses them as he likes without control, and dismisses them when they can pay no more."

Cameron, another notorious quack, began his career in 1809, when he took a back parlour in a street leading from Oxford Street. His method of diagnosis may be inferred from the name by which he was known, to wit "Cameron the Water-Taster," and his plan of treatment was to dose his patients with strong diuretics. The *Medical Adviser* denounces him in unqualified terms, and after explaining that "the secretion of the kidneys may serve as a good indication in many diseases," points out that "to say that any man could come bolt at the disease by merely seeing, smelling, or, as the subject of our memoir does, by tasting it, is nonsense." At the time of his exposure he had "to the disgrace of English common-sense, gone on gulling for fourteen years—absolutely swindling." This class of quack is now extinct in this country, although the writer knows of a charlatan carrying on an extremely lucrative practice in France whose methods are similar.

It would be interesting to take brief glances at some of the other famous quacks who were denounced in the course of this remarkable campaign, but it must suffice to refer in a few sentences to one other only. This is "Doctor" Lamert, who, among his many devices of advertising himself, anticipated the proverbial plan of Bob Sawyer. Lamert used to attend the theatre almost every night, and his servant contrived to call him out several times during the evening, to the no small annoyance of the audience, for those interruptions always happened when some interesting part of the play was going on. "Dr. Lamert is wanted in a minute" would be bellowed out, and up jumped the quack, while the gaping galleries would say, "What a practice Dr. Lamert must have to be sure!" It is interesting to note that this quack practised at least twelve years before the "Pickwick Papers" were written, and curiously enough "Doctor" Lamert, like Bob Sawyer, exercised his profession at Bristol.

It is evident that the views of the editor of the *Medical Adviser* ran contrary to those of the publishers, for before the campaign had run its weekly course for a year there appeared the following announcement. "The most notorious quack in England was apologised to in a humiliating manner. When the quack was mentioned by us he proceeded to the publishers, purchased all the numbers (nearly 2,000) then remaining, engaged Messrs. Knight and Lacey to cut out the paragraph from the stereotyped plates of the number, and employed emissaries to purchase every copy of it to be found in London or its suburbs." In future issues it is evident that the publishers continued to exercise a restraining influence on the editor, and at the end of eighteen months the campaign, and, in fact, the journal in its original form, ceased to exist and the following notice was issued: "When the work commenced it was placed in the hands of Messrs. Knight and Lacey, two industrious young men, and their exertions on its behalf were compensated by its extensive circulation. The profits arising from the sale of the work operated so strongly upon them that they endeavoured by every means in their power to possess themselves of its copyright, and so far succeeded that they *believed* themselves its owner. With this impression they regulated its conduct as to the quality of the matter inserted. Alas! Where is the man who does not first consult his own interest before that of the public? Messrs. Knight and Lacey became acquainted with the quacks. Some of them presented them with haunches of venison, gave them dinners, and gave them wines. It was no wonder, therefore, that articles which were written by the editor against such worthy men should be cut out of the work. Others of the quacks, fancying that they had them in their power and thirsting for revenge, attacked them with the weapons of the law and threatened them with all its thunderbolts. No wonder, then, that men who esteemed themselves as they did should bow down, truckle, and write public apologies to those who threatened them." Thus ended the most vigorous attack upon quackery that has ever been undertaken in this country.

